# Comparison of Testing Methods for Evaluating the Resistance of Alkali-Activated Blast Furnace Slag Systems to Sulfur Dioxide

**DOI:** 10.3390/ma15041344

**Published:** 2022-02-11

**Authors:** Petr Hrubý, Lukáš Kalina, Vlastimil Bílek, Sarka Keprdova, Jiří Másilko, Iveta Plšková, Jan Koplík, Libor Topolář

**Affiliations:** 1 Faculty of Chemistry, Brno University of Technology, 61200 Brno, Czech Republic; kalina@fch.vut.cz (L.K.); bilek@fch.vut.cz (V.B.J.); masilko@fch.vut.cz (J.M.); koplik@fch.vut.cz (J.K.); 2 Faculty of Civil Engineering, Brno University of Technology, 60200 Brno, Czech Republic; keprdova.s@fce.vutbr.cz (S.K.); plskova.i@fce.vutbr.cz (I.P.); libor.topolar@vutbr.cz (L.T.)

**Keywords:** alkaline activation, blast furnace slag, sulfur dioxide, corrosion resistance, non-destructive testing, degradation characterization

## Abstract

Alkali-activated systems (AAS) represent an ecologically and economically sustainable inorganic binder as an alternative to ordinary Portland cement (OPC). One of the main benefits of AAS is their durability in aggressive environments, which can be equal or even better than that of OPC. In this paper, the influence of the type of alkaline activator in alkali-activated blast furnace slag (AAS) in terms of resistance to sulfur dioxide corrosion was investigated. The durability testing process was based on the CSN EN ISO 3231 standard and simultaneously compared with mortar samples prepared by using Blastfurnace cement CEM III/A 32.5R. The degradation progress was evaluated by employing several different methods such as observing the compressive strength development, weight change evaluation, non-destructive testing methods like ultrasound or impact echo technique, or visual phenolphthalein technique. Subsequently, fundamental characterization of samples by the XRD method was performed during the degradation test. The obtained results indicate that none of the testing methods used could be prioritized over others to determine the resistance of AAS against the action of sulfur dioxide. For this reason, the durability testing of AAS remains an issue, and the development of specific standards considering the behavior of AAS seems necessary.

## 1. Introduction

Ordinary Portland cement (OPC) is one of the most used construction materials in the world. Unfortunately, its production is associated with huge emissions of carbon dioxide—since the production of one tone of OPC is related to the formation of approximately one tonne of CO_2_—as well as significant demands for energy [1]. Overall, cement consumption in 2018 was 4.1 billion tons [2]. The production of this amount of cement contributed to worldwide CO_2_ emissions with the non-negligible proportion of 5–8% [3]. Thus, the production of alternative binders with a lower carbon footprint is desirable. 

One suitable candidate is alkali-activated materials (AAMs), for which CO_2_ emission reduction was estimated to be approximately 50 to 100% per ton of produced binder [3]. AAMs are manufactured through chemical reactions between the reactive aluminosilicate source, typically formed as an industrial by-product (blast furnace slag, fly ash, etc.), and the alkaline activator, which is usually an aqueous solution of alkali hydroxide, silicate, or carbonate [4,5]. In the alkaline activation of the blast furnace slag, the reaction products similar to hydrated OPC are formed, such as calcium silicate hydrate (C-S-H), but with incorporated tetrahedral aluminate units (C-A-S-H) and lower CaO/SiO_2_ ratios [6]. Besides the environmental aspects, the alkali-activated blast furnace slag (AAS) shows higher resistance to aggressive environments like acidic solutions [7,8], sulfates [9], chlorides [7], or elevated temperatures [10] compared to the OPC. On the other hand, long-term verification in service life, as well as legislative bases, are missing. 

Nowadays, the atmosphere contains other corrosive gases besides carbon dioxide, like the SO_x_ from burning fossil fuels, petrochemical industries, chemical industries, or metallurgical production. Long-term exposure of concrete to high sulfur dioxide levels is known to produce a series of undesirable chemical reactions causing irreversible deterioration of concrete properties [11]. There are few studies that deal with the characterization of AAS resistance to sulfur dioxide, since the studies published so far [6,12,13,14,15,16,17] are focused on degradation tests using acidic leaching in sulfuric acid. The resistance of both cementitious and alkali-activated binders to the action of sulfuric acid depends mainly on the total CaO content in the binder, as it represents a potential target for the transformation into gypsum and/or ettringite. These newly formed phases have limited structural strength and significantly larger volume than the original reactants, thereby causing expansion and damage to the internal structure [17]. On the other hand, the newly formed gypsum phase can also fill the pores, thus inhibiting further corrosion due to the reduction of porosity and permeability, respectively [5]. The corrosion mechanism of AAS pastes exposed to high concentrations of sulfuric acid (pH = 1) is presented by Allahverdi et al. [18]. The process starts with an ion-exchange reaction (the cation compensating the negative charge of the framework is replaced by H^+^), then the reaction between the calcium and sulfate anion takes place, resulting in the formation of gypsum crystals inside the structure.

Many research studies have demonstrated better AAS resistance to acidic attack compared to OPC due to such facts as (i) different nature of their hydration products, (ii) lower initial permeability, (iii) lower Ca content. Hydrated cement paste contains—besides the C-S-H gel (with high CaO/SiO_2_ ratio)—calcium hydroxide and calcium sulpho-aluminates. The decalcification of these phases leaves a porous corroded layer. Contrarily, the AAS with a coherent A-S-H gel layer is sustained even after decalcification has taken place. This can prevent further ingress of the corrosive medium into the structure and contribute to higher acidic resistance of AAS [7,8,19]. 

There are few techniques that characterize the progress of degradation reactions under the action of sulfuric compounds. However, there is no universal technique useful regardless of the binder or structure type. The loss of mass seems to be a suitable indicator of degradation reactions in general, but Bernal et al. [8] and Lloyd et al. [20] stated reprehensions and suggested the determination of the depth of the corroded layer as a more representative indicator. Nevertheless, [14] expressed doubts over the depth of the lost alkalinity indicator due to its high dependence on the initial alkalinity. Overall, finding a universal technique for the characterization of degradation reactions under the action of sulfuric compounds is difficult [6,12]. The selection of the technique is crucial for proper characterization of degradation degree. 

The determination of compressive strength is the most commonly used technique; it is well known and standardized. The interpretation of data acquired is quite easy and clear. On the other hand, it is sensitive to dimensional and weight changes and/or surface defects like micro cracks or to the presence of spalling parts. Those can distort? the results [14]. The cross-section dimension characterization is useful when the expansion and shrinkage mechanisms take place. The degradation of material can also be captured by means of visual analysis. The main drawback of this technique is significant subjectivity of evaluation as well as the difficulty of exact characterization. Contrary to these drawbacks it allows fast and simple evaluation of macroscopic changes (cracking, spalling, coloration, …) without additional costs [12]. The weight change is useful indicator when the corroded product is separated from the mortar surface and/or dissolved in the solution. The utilization of mass loss indicator can be misleading for concrete if the spalling of large aggregates takes place [14]. The loss of alkalinity determination is extremely beneficial indicator regarding the characterization of carbonation processes and characterization of possibility of steel reinforcement corrosion. The loss of alkalinity is widely used indicator for macroscopic differentiation of affected and non-affected area by means of pH change. However, it is dependent on initial alkalinity of the system. The change of pH of pore solution may not match the changes in phase composition [14,20]. The usage of XRD analysis allows the characterization of phase changes of crystalline products but most of hydration products are amorphous or semi-amorphous which reduces the benefit of this technique. The SEM is used for microstructural characterization. Using the energy dispersive analyser, the SEM-EDS allows the characterization of the elemental composition thus the calculation of changes in C/S ratio etc. The main drawback is small analysed area thus it cannot reflect the properties of the whole sample. The effect of interaction volume of electron beam is not negligible either [12,14].

This paper deals with the determination of sulfur dioxide resistance of various AAS as well as the cementitious system using destructive and non-destructive techniques. The goal is to verify the suitability of these techniques for the characterization of degradation progress. The novelty of this paper is based on the use of non-destructive techniques such as impact echo and ultrasound to characterize the ongoing changes. The elemental combustion analysis for the determination of the sulphur content is not commonly used technique for these purposes either. Moreover, the applicability of current standards for the SO_2_ resistance testing for AAS was tested and verified. Beside these characterization criteria the effect of alkaline activator type on the resistance to the action of sulfur dioxide was established. 

## 2. Materials and Methods

### 2.1. Materials

Alkali-activated blast furnace slag (BFS) systems were prepared using slag with the Blaine fineness of 400 m^2^∙kg^−1^ (ArcelorMittal Ostrava, s.r.o., Ostrava, Czech Republic). Its phase composition was determined by means of X-ray powder diffraction (XRD) using the Rietveld method with internal standard (fluorite). The BFS showed an amorphous content of about 70.5%. The main crystalline phases identified were akermanite (19.8%), calcite (6.7%), quartz (2.7%), and merwinite (0.4%). Liquid sodium water glass—silicate modulus (Ms) = 0.5 (Vodní sklo, a.s.., Brno, Czech Republic), sodium hydroxide—50 wt.% solution (Penta s.r.o., Praha, Czech Republic) and sodium carbonate (Inchema s.r.o., Horní Počernice, Czech Republic) were used as alkaline activators. The comparative cementitious binder was prepared using the CEM III/A 32.5R (Horné Srnie cement plant, Cemmac s.r.o., Horné Srnie, Slovakia). The chemical compositions of both BFS and Blastfurnace cement were determined using the X-ray fluorescence analysis (XRF) and it is shown in Table 1.

### 2.2. Preparation and Composition of Mortars

The testing samples were prepared according to the CSN EN 196-1 [21]. The mixing lasted 3 min, during the first 30 s the activator and the slag (or CEM III/A) were mixed using low speed (260 rpm), then sand was added and mixed for other 30 s. Then the high-speed (1250 rpm) mixing took place for 30 s and was followed by wiping the walls of container during 30 s. The final mixing using high-speed for 60 s was done at the end. The laboratory mixer (KitchenAid Robot Artisan 175, Whirlpool Corporation, Benton Harbor, MI, USA) was used for the mixing. The prepared mixture was placed in moulds and compacted on the compacting table for 30 s. The samples were demoulded after 24 h and placed in the water storage. The mortar bars (dimension of 40 × 40 × 160 mm^3^) were stored underwater for up to 28 days at 25 °C before the initialization of the sulfur dioxide resistance testing. The sand to binder ratio in the mortars was 3:1 in terms of mass. The water to binder ratio (w/b) was kept the same (0.45) for all alkali-activated and cementitious systems. The amount of water in the water glass and 50% sodium hydroxide solution was counted into the total w/b. Various alkaline activators: sodium hydroxide (SH), sodium carbonate (SC), and sodium water glass (SWG)—Ms = 0.5 were used for the binder preparation but with constant Na_2_O dosages (6 wt.% per BFS weight). The Na_2_O content in each activator was mathematically calculated and verified by means of acid-base titration with conductometric determination of the equivalence point. Table 2 describes the composition of the binders prepared. The total w/b was the same for all binders but the content of added water differs due to the factors like humidity or the water content in SH or SWG solutions. 

### 2.3. Testing of Resistance to Sulfur Dioxide 

The determination of resistance to humid atmospheres containing sulfur dioxide was performed in accordance with the CSN EN ISO 3231 [22]. The testing samples were exposed to the action of sulfur dioxide, elevated temperature, and humidity in a watertight chamber. The concentration of SO_2_ was equal to 3.375‰. The chamber operated in a cyclic mode. The steps were as follows: (i) closure of the chamber; (ii) insertion of sulfur dioxide through the demineralized water; (iii) heating of chamber up to (40 ± 3) °C for 8 h followed by turning the heating off and opening the door of the chamber; (iv) the cycle ended and started again from Step (i). The corrosion gas chamber HK 800 (Köhler Automobiltechnik GmbH, Lippstadt, Germany) with a volume of 800 L was used for this purpose. One cycle lasted 24 h (8 h in a humid environment containing SO_2_ and then 16 h of ventilation). The samples were then tested after 20 (480 h); 40 (960 h); 100 (2400 h); and 200 (4800 h) cycles to SO_2_ exposition using a number of destructive and non-destructive techniques, which will be further specified. 

### 2.4. Testing Methods

The mortar bars were used for the determination of compressive strength based on the CSN EN 196-1 standard [21] (Desttest 3310, Beton System, s.r.o., Brno, Czech Republic) and depth of the area with decreased pH using the phenolphthalein technique. The phenolphthalein technique was included for the determination of the progress of degradation reactions since these processes are accompanied by the decreased pH of the matrices. The fracture areas after the three-point bending test were treated with a 1% solution of phenolphthalein in ethanol. The treated surfaces were photographed, and the dimension of the degraded (colorless) area was measured and evaluated using the optical analysis method. The measurement of weight change and other non-destructive techniques took place at the same intervals. 

The ultra-sound method was based on the changes of permeability and reflectance of ultrasound waves due to the disintegration of the tested material. The ultrasound passage technique is based on the initialization of the short high-frequency pulse generated by the source, and the time of passage of longitudinal ultrasound waves through the tested sample was then determined [23]. A Pundit Plus set with two 54 kHz probes (transmitting and receiving) was used for the measurement of ultrasound pulse passage. The usage of a binding element (thin layer of plasticine applied on the surface of both probes) was necessary to secure a better transfer of the signal between the probe and the sample. The time of passage through the acoustic connecting material and the probe is called dead time. Dead time is determined using the standard with a known time of passage [24,25]. The standard had the value of 13.6 µs in our case. Testing samples were stored on the rubber underlayment during the measurement, and the measurement was done perpendicularly to the direction of compaction. The time of passage of longitudinal ultrasound waves through the sample was measured, and the probes were placed on the surface of the sample in identical places. Each sample was measured three times. The measurement was based on the CSN EN 12504-4 (73 1303) [26].

**Figure 1:**
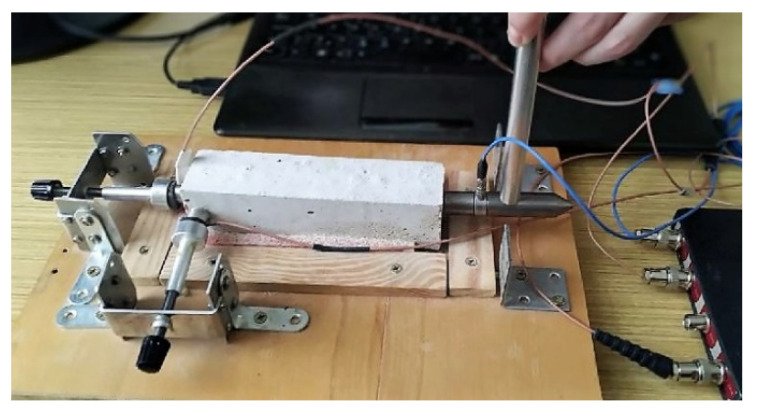
The photo of experimental arrangement during the impact-echo measurements.

The impact-echo technique (resonance method) is based on frequency analysis of the response of a mechanical impulse, which excites harmonic waves in the investigated sample at its own frequency and at higher harmonic frequencies [27,28]. The mechanical pulse is generated by the impact of the hammer on the surface of the sample. This impact creates low-frequency stress sounds, which are spread through the structure, and reflect the internal defects and external pores [29]. The resulting response in the form of surface waves is detected using the piezo-electric detector of a DAKEL MIDI type (ZD Rpety-Dakel, Horovice, Czech Republic). This detector was attached to the sample using plasticine and pressed by securing the element in the measuring device. The measurement of impact-echo was done under the same conditions each time. The detection of the resulting response of the sample was done using the two-channel oscilloscope TiePie Engineering Handy Scope HS3 (TiePie Engineering, Sneek, The Netherlands) with a 16-bit resolution. The evaluated parameter of impact-echo measurement was the shift of the dominant frequency. The dominant frequency was obtained from the frequency spectra analysis. The obtained dominant frequencies were in the interval 3.5–5.2 kHz for all specimens. The detected signal was converted from the time to frequency parameter using the fast Fourier transformation [30]. The measurement was done according to the CSN 73 1372 [31]. The transducer arrangement is shown in Figure 1. The probes were attached to opposite faces of the longer side of beam (160 mm). The same experimental arrangement was used for the ultra-sound testing.

Alkali-activated and cementitious pastes (w/b = 0.35) underwent the same testing procedure and were used for the XRD analysis since the presence of siliceous aggregate caused a reduction in the intensity of the monitored peaks.

The XRD analyses were carried out using the powder diffractometer (Empyrean PANalytical, Malvern Panalytical Ltd., Malvern, UK) with the following parameters: Cu Kα_1_ radiation (1.54059 Å) tube current 30 mA and voltage 40 kV; scan axis gonio; step size 0.01313° 2Θ time per step 96 s, scan range 4.5–90° 2Θ. The results were evaluated using the software HighScore plus. The semi-quantitative analyses were done due to the amorphous content in the samples. The content of sulfur was determined using the elemental combustion analyzer for C, S determination (G4 Icarus, Bruker, Billerica, MA, USA). The chemical composition of raw materials was determined using the XRF analyzer (Vanta VCR, Olympus-IMS Ltd., Quebec City, QC, Canada) with the following parameters: voltage 40 kV, output 4 W, the anode material Rh, detector resolution ≤138 eV, current 200 µA.

## 3. Result and Discussion

### 3.1. Compressive Strength Evaluation

**Figure 2:**
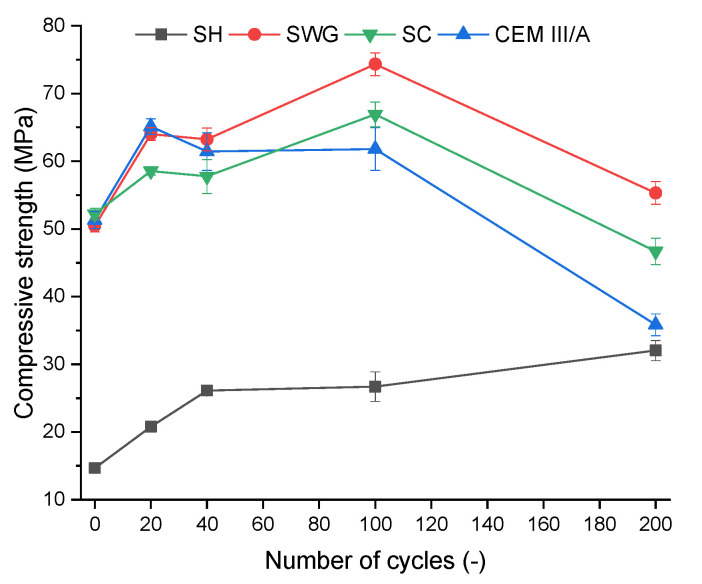
Change of compressive strength as a function binder type and number of cycles. (SH – sodium hydroxide activated blast furnace slag; SWG—sodium water glass activated blast furnace slag; SC—sodium carbonate activated blast furnace slag; CEM III/A—blast furnace slag cement).

The compressive strength is one of the most often used indicators to characterize the progress and impact of degradation reactions. Figure 2 shows the compressive strength development depending on the number of cycles for various binder systems. All tested binder systems showed similar trends of improving the mechanical properties up to 100 cycles. This is related to the reactions of latently hydraulically active BFS that take place since the BFS is present as well as sufficient alkaline pH. The latent hydraulic activity is an alternative to the pozzolanic reactions which are known to be quite slow and dependent on the portlandite content in the OPC [32,33,34]. In case of the AAS the initiator is not the portlandite but the alkaline solution. The AAS are known to have fast and rapid setting as well as compressive strength development, still the hydration reaction continues further in time and reflects in overall the properties like microstructure, compressive strength or phase composition [5]. It seems that the proceeding hydration reaction competes with the degradation reactions during the first 100 cycles and they influence each other. The action of degradation effects prevails after 100 cycles and the contribution of hydration reactions fails to compensate for the degradation effect. The SH activated system seems to be the exception since its resistance to the action of the SO_2_ was the highest and the improvement of compressive strength due to the hydration reactions was observed.

In the case of SH, the increase of compressive strength of about 20% was observed between 100 and 200 cycles, whereas rapid deterioration of compressive strength was found for all other binders. The drop of compressive strength by about 25, 30 and 42% was observed for SWG, SC, and CEM III/A, respectively, between 100 and 200 cycles. The deterioration of mechanical properties of AAS can be attributed to the formation of micro and macro cracks due to repeated cycles of drying and wetting. It has been shown that AAS are sensible to shrinkage, resulting in the formation of cracks and deterioration of overall properties. This also limits the expansion of their industrial utilization [35,36,37].

The shrinkage occurs as the result of the following independent factors: (i) change of humidity, (ii) chemical reactions, and (iii) physical interactions of nanoparticle surfaces of hydration products and pore solution [38]. There are several shrinkage mechanisms (autogenous and chemical, plastic, drying, or decalcification shrinkage [5]), while the last two mentioned have dominant effects in the present study. The drying shrinkage is considered to be the most dangerous one and cannot be eliminated during the service life of construction segments because the changes in humidity will always take place [37]. AAS suffers from drying shrinkage several times more than ordinary Portland cement, not only due to the different pore size distributions, but also due to the nature of hydration products [37,39,40,41]. The decalcification or carbonation shrinkage contributes to the deterioration of physic-mechanical properties of the binders as the cycling regime allows the carbonation as well as the decalcification reactions, which was also confirmed by the presence of carbonation products and gypsum using the XRD. The decalcification of the CSH phase is related to the changes in its structure. As the calcium ions are eliminated from the CSH, the polymerization reactions of resting silicates occur. This leads to the shrinkage of the matrix along with the formation of micro-cracks [42]. The effect of decalcification shrinkage is less significant for the AAS than for the OPC as presented by Komljenović et al. [43] due to the absence of portlandite, higher degree of polymerization of CSH, and incorporation of alumina units into the CSH, and the formation of a stable passivation layer of Si-gel. However, they are more prompt to the carbonation reaction because there is no portlandite that preferably carbonates to calcium carbonate, thus filling the pores and retarding further penetration of CO_2_ [44]. Under the experimental conditions of SO_2_ cycling, all the mentioned shrinkage mechanisms can coexist and influence each other.

The shrinkage of AAS is dependent on the activator type as well as its dosage, curing conditions, or the addition of shrinkage-reducing admixtures [5]. The sodium hydroxide-activated systems show the lowest shrinkage in contrary to the water glass-activated systems. The presence of SiO_2_ in the activator plays an important role in the mechanical properties and shrinkage. An increase of shrinkage with increasing silicate modulus was observed [45,46]. This can explain the non-deterioration of mechanical properties of SH-activated slag due to the cycling regime since the SH-activated systems are the least susceptible to shrinkage. However, Ref. [46] presented the lowest drying shrinkage when the sodium carbonate was used as an activator and had shrinkage comparable to the OPC. The usage of sodium hydroxide was related to three times higher shrinkage than the usage of OPC, and alkali-activation using the liquid sodium water glass showed ten times higher shrinkage.

Puertas et al. [47] found that SWG-activated systems were more prompt to the effect of carbonation than the SH-activated one. While the SWG-activated system showed a decrease in compressive strength and an increase in porosity, the SH-activated one provided practically the opposite results, i.e., an increase in strength and an increase in matrix compactness. The different length of the silicate chain and the Ca/Si ratio explains the different behavior. The silicate chain is longer, and the Ca/Si ratio is lower (≈0.8 vs. 1.2) when SWG is used as an activator. The higher Ca/Si ratio in SH-activated systems helps to precipitate CaCO_3_ and heals the pores (the self-healing ability was also presented in [48]). A higher rate of carbonation was observed for SC than for SWG-activated systems. In the case of SWG, the silicate modulus also plays a role. A higher rate of capillary sorption of carbon dioxide was observed for lower Ms.

The formation of expansion products like gypsum, the presence of which was detected using the XRD (see Section 3.5), also contributes to the deterioration of mechanical properties of the samples; however, the effect of shrinkage seemed dominant. To eliminate the effect of shrinkage on the physical-mechanical properties, immersion in sulfuric acid solution can be used for the simulation of SO_2_ corrosion resistance. These tests were used in several studies [6,14,15,16], where authors stated similar deterioration of compressive strength due to the immersion of various AASs in the sulfuric acid. The deterioration of compressive strength is explained by the formation of gypsum associated with the volume expansion and the formation of defects in the structure. This opens the space for further and more rapid acid penetration, which causes the increase of degradation rate in time.

The results obtained indicate the coexisting effect of drying shrinkage, carbonation, and the action of SO_2_ on the deterioration of mechanical properties, and the individual contributions cannot be fully distinguished. To conclude, the SH-activated system showed no signs of deterioration of compressive strength, which corresponds well with the results of the non-destructive techniques, where the lowest increase of time passage, as well as the highest resonance frequencies, were observed. Still, it seems to be the result of the lowest inclination to the shrinkage over the superior resistance against the effect of SO_2_. Moreover, the results of the phenolphthalein technique (Section 3.4) indicate that the SH-activated system showed the lowest resistance against the chemical attack (action of sulfur dioxide and carbonation).

### 3.2. Ultrasound and Impact-Echo Evaluation Techniques

**Figure 3:**
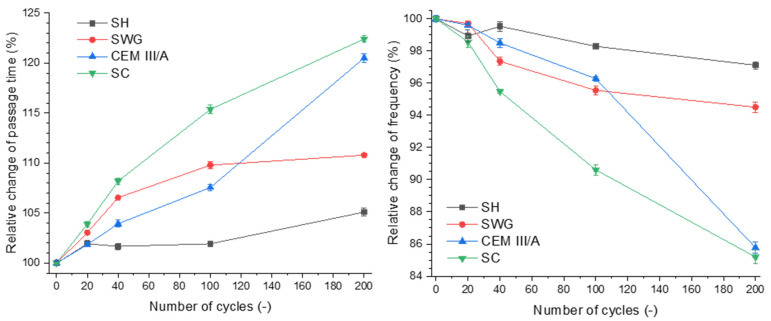
Relative change of passage time (**left**) and frequency (**right**) as a function of binder type and number of cycles. (SH—sodium hydroxide activated blast furnace slag; SWG—sodium water glass activated blast furnace slag; SC—sodium carbonate activated blast furnace slag; CEM III/A—blast furnace slag cement).

Figure 3 shows the dependence of relative change of passage time on the number of cycles and the relative change of frequency.

The increase of passage time of the longitudinal ultrasound waves through the sample can be assigned to the formation of internal disintegrations. This is related to the extension of the passage of ultrasound waves from one probe to the other [23,25]. The passage times were prolonged with the increasing number of cycles for all tested binders. Secondly, the resonance frequency was decreasing as the structural changes caused by degradation processes were taking place.

Regarding the presented results, it seems that both ultrasound and impact-echo techniques can be considered as substitutable methods for characterizing the progress of degradation and can be used as an indicator of degradation of the material, especially since they can be used simultaneously during the whole degradation process. The selected non-destructive techniques are complementary, and their correlation was confirmed. The SH-activated system showed changes of both the passage time and the resonance frequency of approximately 5%; thus, they were almost negligible. This indicates that these non-destructive techniques are not able to catch the minor changes associated with the formation of gypsum without expansive cracking. This is contrary to the SC-activated system and the CEM III/A, where changes of ~20% were observed. The changes of both passage time and resonance frequency reflected the formation of cracks as a result of drying shrinkage.

### 3.3. Weight Change Evaluation

**Figure 4:**
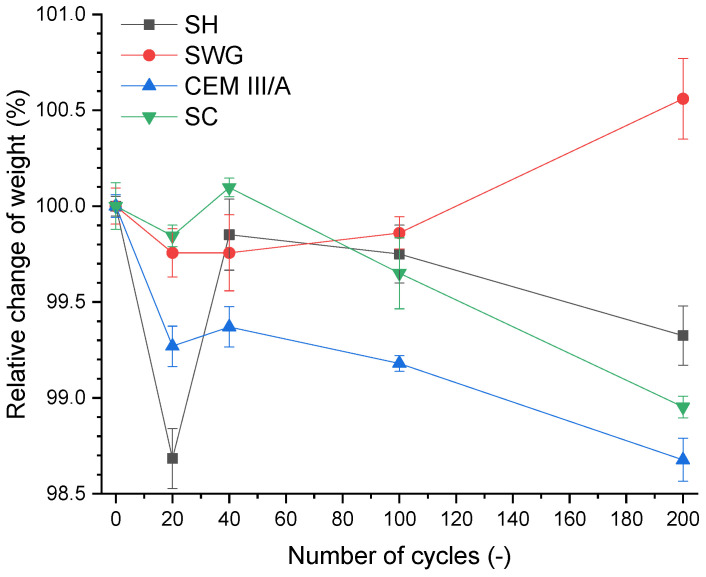
Relative change of weight as a function of binder type and number of cycles. (SH—sodium hydroxide activated blast furnace slag; SWG—sodium water glass activated blast furnace slag; SC—sodium carbonate activated blast furnace slag; CEM III/A—blast furnace slag cement).

The weight change of the specimen as a loss of the initial weight is a widely used indicator for the assessment of deterioration of mortars subjected to an aggressive environment. The 28-day cured samples were used as a reference, and they were marked as zero cycles. The course of weight change during the SO_2_ cycling is presented in Figure 4.

There were no significant changes (±1.5%) in the weight of the mortars for the tested binders during 200 cycles. This corresponds to the results presented in [12], where the immersion in sulfuric acid (1–5% concentration) was used to determine the degradation resistance, and no rapid changes were noticed. The weight change evaluation based on a higher loss of mass was also presented due to the immersion in sulfuric acid. The loss of mass (less than 10%) due to the immersion in 10% sulfuric acid was stated in [16] for alkali-activated systems based on the mixture of fly ash and blast furnace slag. A slightly higher loss of mass due to the immersion in 1% H_2_SO_4_ was observed in Gu et al. [14]. They reported a loss of mass of approximately 40% after 500 days of immersion. The effect of repeated wetting/drying on weight changes seemed to predominate over the sorption of SO_2_ alone in view of the obtained results.

### 3.4. Visual Appearance/Phenolphthalein Technique

**Figure 5:**
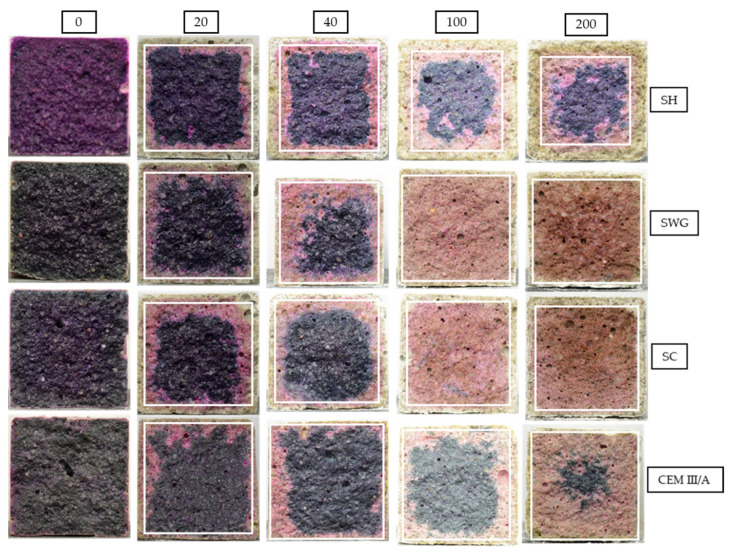
The cross-sections of mortar samples sprayed with phenolphthalein solution after 0, 20, 40, 100, and 200 cycles in SO_2_ chamber. (SH—sodium hydroxide activated blast furnace slag; SWG—sodium water glass activated blast furnace slag; SC—sodium carbonate activated blast furnace slag; CEM III/A—blast furnace slag cement).

**Figure 6:**
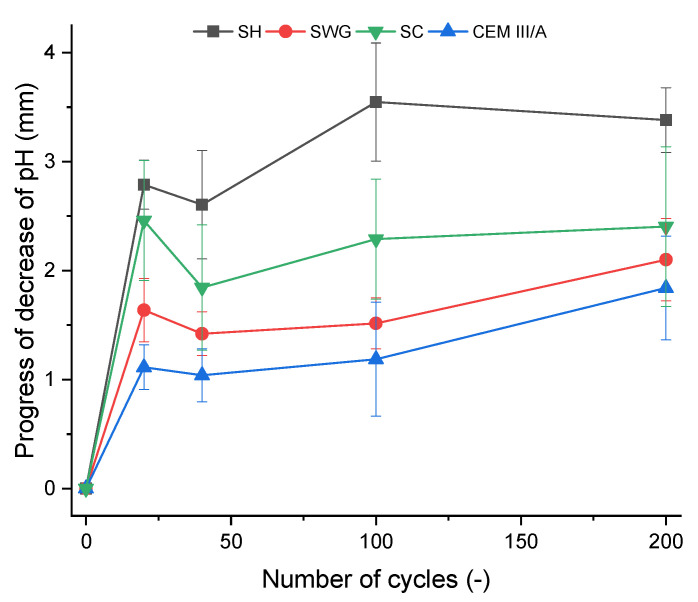
The depth of affected cross-section area of the mortar samples as a function of number of cycles in the SO_2_ chamber. (SH—sodium hydroxide activated blast furnace slag; SWG—sodium water glass activated blast furnace slag; SC—sodium carbonate activated blast furnace slag; CEM III/A—blast furnace slag cement).

The phenolphthalein technique is often used to characterize the progress of degradation reactions accompanied by the decrease of the pH of matrices. This is reflected by the color change of the indicator from purple-red to colorless. The photographs of phenolphthalein-treated fracture surfaces are presented in Figure 5, which contains the selected photos representing the sample the closest to the mean value of tested samples. The graphic representation of determined area (the average value of the thickness of the layer) with lost alkalinity is shown in Figure 6. Three samples were evaluated for unique binders in each interval.

The greatest pH decrease was observed for the SH-activated system. It resulted in the areas with lost alkalinity being increased by ~62, 71, and 54% compared to those measured for SWG, SC-activated systems, and the cementitious one after 200 cycles. This indicates that the highest modifications in chemical composition associated with the changes in pH took place in the SH-activated system (this corresponds to the lowest buffering capacity of the SH-activated system). On the other hand, the other tested binders did not undergo such significant changes. This trend is exactly opposite to the previously discussed results of compressive strength development or the non-destructive techniques. This seems to be related to the elimination of the effects of shrinkage and cracking, thus in our case, the results of the phenolphthalein technique should correspond to and reflect the greatest action of the SO_2_ and carbonation processes. The more noticeable increase of the loss of alkalinity started after 100 cycles and continued further as a reaction to carbonation and decalcification (gypsum formation). This corresponds to the XRD results where the relevant changes in phase composition start after 100 cycles.

The phenolphthalein technique can distinguish areas with pH values lower than 8.2, but its applicability is limited to comparing the loss of alkalinity of one binder over time. Different materials, however, cannot be compared. The test results are strongly influenced by the initial alkalinity, and therefore, the test cannot be considered a universal technique [14]. On the other hand, Lloyd et al. [20] reported the benefits of the phenolphthalein technique contrary to the weight change determination when comparing various AASs. The weight of the samples remained the same, indicating that the dissolution of the binder took place, but the surface layer was porous and showed low strength.

The photos in Figure 5 show changing coloration of the binders’ systems regardless of the treatment by phenolphthalein. The color changes accompanying the hydration of the AAS had not yet been fully understood. The blue/green coloration can be explained by the presence of polysulfide species. The sulfur in the blast furnace slag is in the form of sulfides, due to the reducing atmosphere during the production process of slag as a by-product of iron production. The reducing conditions can be found during the hydration of AAS as well, since the presence of HS^–^ was observed [49]. The sulfide anion can undergo gradual oxidation to form a polysulphide radical. The S_2_^−^ ion is a green chromophore and the S_3_^−^ is then a blue chromophore. This can at least partially explain the color changes during the hydration of AAS. However, the influence of transition metal complexes, especially ferrous compounds, remains possible [50,51]. As the hydration reaction continues, the intensity of coloration increases. On the other hand, the decrease of intensity to white or yellow is probably related to the oxidation of sulfide and ferrous species [51]. The depth of the oxidated layer after 200 cycles increases for tested binders in the following order CEM III/A < SWG < SC < SH.

### 3.5. X-ray Powder Diffraction (XRD) and Elemental Analysis

**Figure 7:**
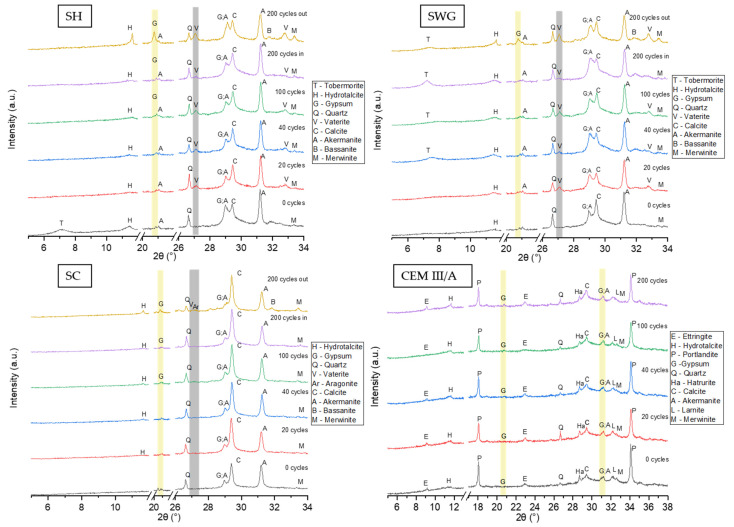
The XRD patterns for all tested binders as a function of number of cycles in SO_2_ chamber. (SH—sodium hydroxide activated blast furnace slag; SWG—sodium water glass activated blast furnace slag; SC—sodium carbonate activated blast furnace slag; CEM III/A—blast furnace slag cement).

To describe the phase changes within the tested binder systems, X-ray powder diffraction analysis was conducted semi-quantitatively. Figure 7 shows relevant XRD patterns for tested binders. The results of the phenolphthalein technique were used for the selection of the area to be analyzed by XRD. The inner and outer layers were not distinguished during the initial 100 cycles regarding the fact that the area with lost alkalinity was not persuasive. Thus, in this case, the sampling was conducted as a cross-section from the sample surface to the center. Contrary to the sampling after 200 cycles where the inner and outer layers varied. The outer layer was selected as the one with lost alkalinity (colorless); meanwhile, the inner one remained purple-red and was taken from the center of the paste sample. For the SH-activated samples, gypsum was the reaction product of the interaction of sulfur dioxide and calcium compounds present in the binder, which occurred after 100 cycles. Much higher concentrations of gypsum could be observed in the outer layer since the penetration of SO_2_ started at the surface of the sample. The presence of calcium sulfate hemihydrate was also observed in the outer layer of the sample after 200 cycles. The applied cycling procedure, in addition to the action of SO_2_ itself, also stimulates carbonation. Thus, the carbonation products, like calcite and vaterite, were detected. Especially the presence of vaterite indicates that the carbonation process took place, since it is a metastable compound that is formed during the reaction between atmospheric carbon dioxide and calcium ions and then converted to a more stable form—calcite [52]. Calcite was detected even in the samples without treatment in the SO_2_ chamber, as it was already present in slag. The XRD analysis of SWG-activated slag provided similar results. The presence of gypsum was detected only in the outer layer of the samples after 200 cycles. The phase composition of AAS prepared using the SC activator showed the presence of gypsum after 100 cycles.

The gypsum was observed as the main reaction product of degradation reactions because of the action of sulfuric acid [12,13,15,16] or sulfur dioxide [11,53]. The cementitious system did not show any relevant changes in crystalline phase composition throughout the testing interval. Still, the decalcification of CSH could take place due to both the action of SO_2_ and CO_2_ and the formation of the corresponding reaction products incorporating the calcium ions originating from the binder gel.

The quantification of gypsum formed due to the action of sulfur dioxide was conducted by means of elemental analysis using a combustion analyzer. The results are presented in Table 3. While the results of phenolphthalein indicate the greatest chemical damage in the SH-activated system, the results of the combustion analysis show the lowest increase in sulfur; thus, the lowest amount of gypsum formed. The internal part of the sample did not show any increase of sulfur content (3%) in contrast to the outer part, where an increase of sulfur content of approximately four times was observed when compared to the sulfur content before cycling and after 200 cycles. Still, that increase was the lowest from all alkali-activated systems tested (SWG-activated showed the increase of about six times and SC-activated one of about eight times). An increase of sulfur content in the internal parts of SWG and SC-activated systems was also observed (217 and 192%) contrary to the SH-activated one. The cement was above all AAS, with only a 45% increase. However, the initial sulfur content in cement was already three times higher than that in AAS. The results obtained for CEM III/A correlated well with the phenolphthalein technique since the loss of alkalinity was the lowest; actually, there was almost none (±1.5 mm), and from XRD data, no relevant changes in phase composition were observed due to the action of SO_2_. The increase of sulfur content in the binders should correlate with the amount of gypsum or sulfuric compounds like bassanite or ettringite.

## 4. Conclusions

The resistance of different types of alkali-activated blast furnace slag systems to the action of sulfur dioxide was studied in this paper. At the same time, the suitability of various destructive and non-destructive techniques for the determination of the process and the degree of degradation was evaluated. Based on the obtained results, the following conclusions can be drawn:

• The decrease of compressive strength during the standard testing of SO_2_ resistance according to CSN EN ISO 3231 was found for the systems activated by SWG, SC activator, and for CEM III/A. Contrarily, SH-activated BFS systems did not show any decrease in compressive strength.
• Both ultrasound and impact-echo seem to be suitable techniques for the characterization of the progress of degradation. The usage of these methods is extremely beneficial for the evaluation of the state of building structures during service life.
• The phenolphthalein technique indicating the loss of alkalinity of the matrix only showed a correlation with the XRD technique out of all characterization techniques.
• The elemental analysis showed significant increase in sulfur content due to the SO_2_ cycling. The combination of XRD and elemental analysis seems to be suitable for the characterization of undergoing changes in phase composition and for the quantification of formed gypsum.
• The resistance of various AAS against the action of SO_2_ according to the CSN EN ISO 3231 increases in the following order of activators: SC < SWG < SH.
• SH-activated blast furnace slag showed the highest overall resistance to the action of SO_2_ of all tested binders.
• Currently used standards are not applicable to the AAS. The wetting/drying cycles probably had more negative effects on the properties of tested binders than the effect of SO_2_. Therefore, the more appropriate method for the evaluation of SO_2_ resistance, which would take into account the characteristics of AAS, needs to be applied.
• Further research should focus on the profound characterization of undergoing processes using other instrumental techniques. An innovative experimental design for the determination of the SO_2_ resistance suppressing the unsatisfactory aspects of the current one should be designed.

## Figures and Tables

**Table 1:** Chemical composition of BFS and Blastfurnace cement as determined by X-ray fluorescence.

	Chemical Composition in wt.%										
	CaO	SiO_2_	MgO	Al_2_O_3_	SO_3_	TiO_2_	K_2_O	MnO	Na_2_O	Fe_2_O_3_	LOI
BFS	37.0	39.4	8.6	8.1	1.4	0.3	1.2	0.9	0.4	0.7	2
CEM III/A	45.6	31.6	5.8	7.4	3.3	0.4	0.8	0.6	0.3	1.4	2.8

**Table 2:** Composition of unique alkali-activated systems and the cementitious mixture expressed in wt.%.

	BFS	CEM III/A 32.5R	Sand	50% NaOH	Sodium Water Glass (Ms = 0.5)	Na_2_CO_3_	H_2_O
SH	22.2	–	66.5	3.4	–	–	7.9
SWG	22.0	–	66.1	–	4.6	–	7.2
SC	22.0	–	66.2	–	–	2.3	9.5
CEM III/A	–	22.5	67.4	–	–	–	10.1

**Table 3:** The results of elemental analysis for unique binders after 200 cycles.

	Sulfur Content in wt.%		
	0 Cycles	200 Cycles In	200 Cycles Out
SH	0.411 ± 0.039	0.423 ± 0.046	2.201 ± 0.031
SWG	0.362 ± 0.004	1.150 ± 0.020	2.542 ± 0.040
SC	0.319 ± 0.008	0.931 ± 0.011	2.964 ± 0.011
CEM III/A	1.031 ± 0.007	1.491 ± 0.055	

## Data Availability

All the data is available within the manuscript.
